# A Novel Approach to the Management of an Intra-abdominal Abscess: A Case Report and Literature Review

**DOI:** 10.7759/cureus.78022

**Published:** 2025-01-26

**Authors:** Maria F Guevara-Kissel, Kenechukwu Egbuonu, Sebastian Valdivieso, Shamon Gumbs, Max Murray-Ramcharan, Maxwell Kissel, Osti Narayan, Hadley Cadot

**Affiliations:** 1 Department of Surgery, Harlem Hospital/Columbia University, New York, USA; 2 Department of Surgery, Columbia University Vagelos College of Physicians and Surgeons, New York, USA; 3 Department of Internal Medicine, The Brooklyn Hospital Center, New York, USA

**Keywords:** abdominal sepsis, alteplase (tpa), ct-guided drainage, drain output, exploratory laporotomy, gunshot injury to abdomen, intra-abdominal collection, less invasive techniques, major trauma, subphrenic abscess

## Abstract

Exploratory laparotomies for blunt or penetrating trauma often result in significant morbidity. Despite advancements in resuscitation, surgical techniques, and antibiotics, intra-abdominal abscesses remain a serious complication, contributing to poor outcomes and extended hospital stays. Percutaneous computed tomography-guided drainage is the standard treatment for abscesses, offering high success rates and low morbidity. However, its efficacy depends on factors such as abscess location and radiologist expertise. In cases where drainage is inaccessible, open or laparoscopic surgery may be required, which carries substantial risks. In rare situations, administering tissue plasminogen activator (tPA) via an abdominal drain has been shown to resolve abscesses effectively.

This report discusses a 37-year-old male patient with a gunshot wound to the left upper abdomen, resulting in hemoperitoneum, gastric injury, and lacerations to the kidney and pancreas. Following surgical repair and placement of a Jackson-Pratt drain, the patient developed sepsis and a subphrenic abscess that could not be accessed for interventional radiology drainage. After weighing the risks and benefits, tPA was administered via the Jackson-Pratt drain, leading to clinical improvement. This innovative approach may offer an alternative for managing difficult-to-drain intra-abdominal collections, potentially reducing surgical intervention and associated morbidity.

Currently, no large-scale studies or consensus exist regarding tPA use and dosing for abdominal collections, highlighting the need for further research. Insights from intrapleural tPA application could inform its broader use in intra-abdominal treatments.

## Introduction

Laparotomy for trauma patients comes with a significant and persistent challenge: infection. Even with advancements in surgical prophylaxis, infection rates remain alarmingly high, making it the leading cause of death among those who survive beyond the critical first 48 hours post-operation [1-3].

Intra-abdominal infections are particularly prevalent, representing the most common infectious complication in abdominal trauma cases, with incidence rates ranging from 2% to 9% [4]. Despite notable progress in resuscitation techniques, surgical innovations, peritoneal irrigation methods, and antibiotic therapies, intra-abdominal abscesses continue to pose a challenge. These abscesses not only complicate recovery but also contribute to delayed mortality, prolonged hospital stays, and an increased need for reoperations in severe trauma cases, highlighting the ongoing need for improved management strategies.

Percutaneous computed tomography (CT)-guided drainage is widely regarded as the gold standard for managing intra-abdominal abscesses [5]. This article explores the use of tissue plasminogen activator (tPA), a thrombolytic agent that facilitates fibrin clot breakdown by converting plasminogen into plasmin, as a potential alternative treatment in cases where image-guided percutaneous drainage is not feasible.

This case report was presented as a poster at Digestive Disease Week (DDW 2024) in Washington, D.C., on May 19, 2024.

## Case presentation

A 37-year-old man was brought to the Emergency Room after sustaining a gunshot wound to the left upper quadrant of the abdomen. On arrival, the patient was hemodynamically unstable. He was taken to the operating room for exploratory laparotomy. 

**Figure 1:**
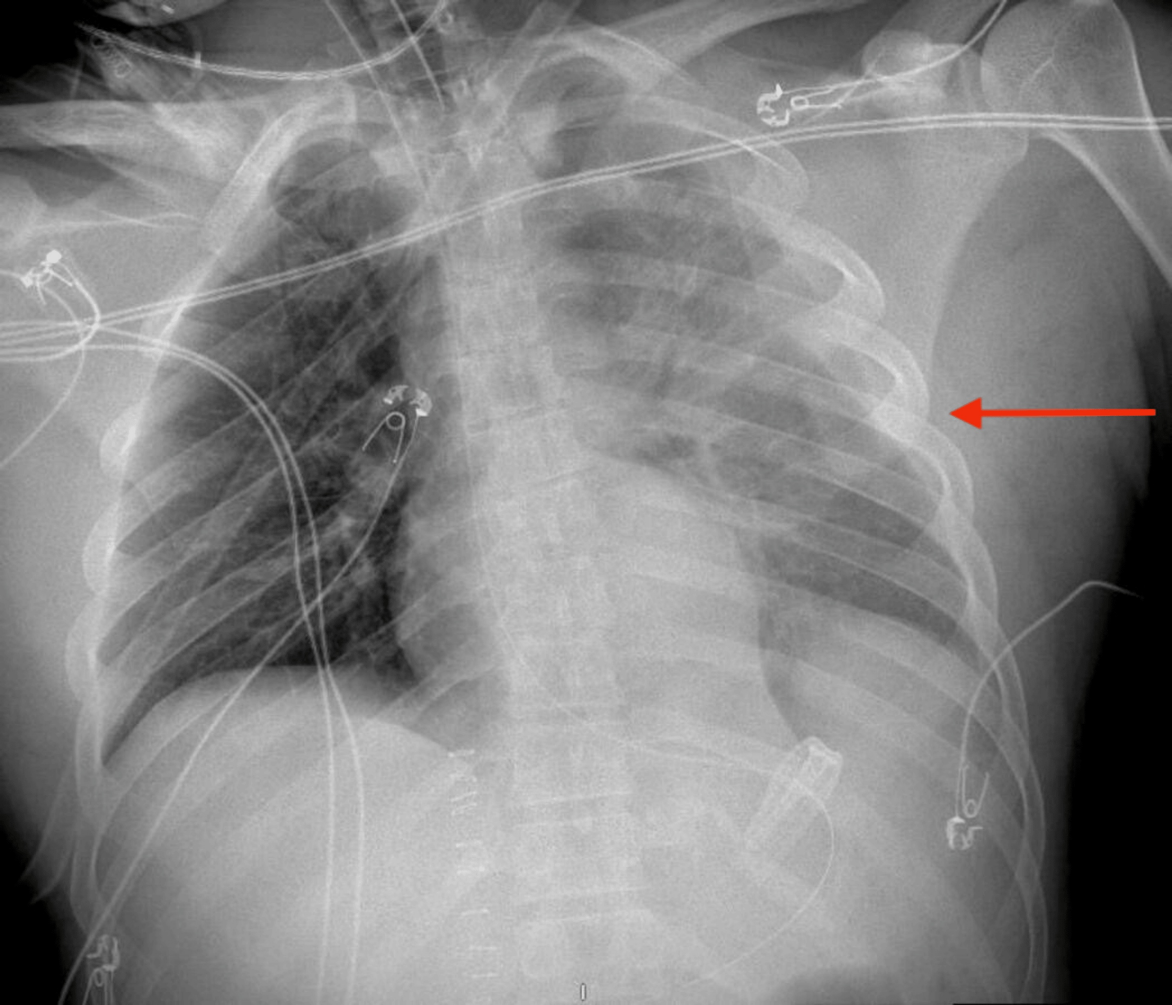
Chest X-ray showing left hemothorax (red arrow).

Intra-operatively, there were hemoperitoneum, gastric injury, kidney laceration, and a pancreatic tail laceration. The stomach injury was repaired; the intra-operative methylene blue leak test was negative after repair. A Jackson-Pratt drain was left in the left upper quadrant near the pancreas. Post-operatively, a chest radiograph revealed a left-sided pleural effusion consistent with a left hemothorax as visible in Figure 1. 

**Figure 2:**
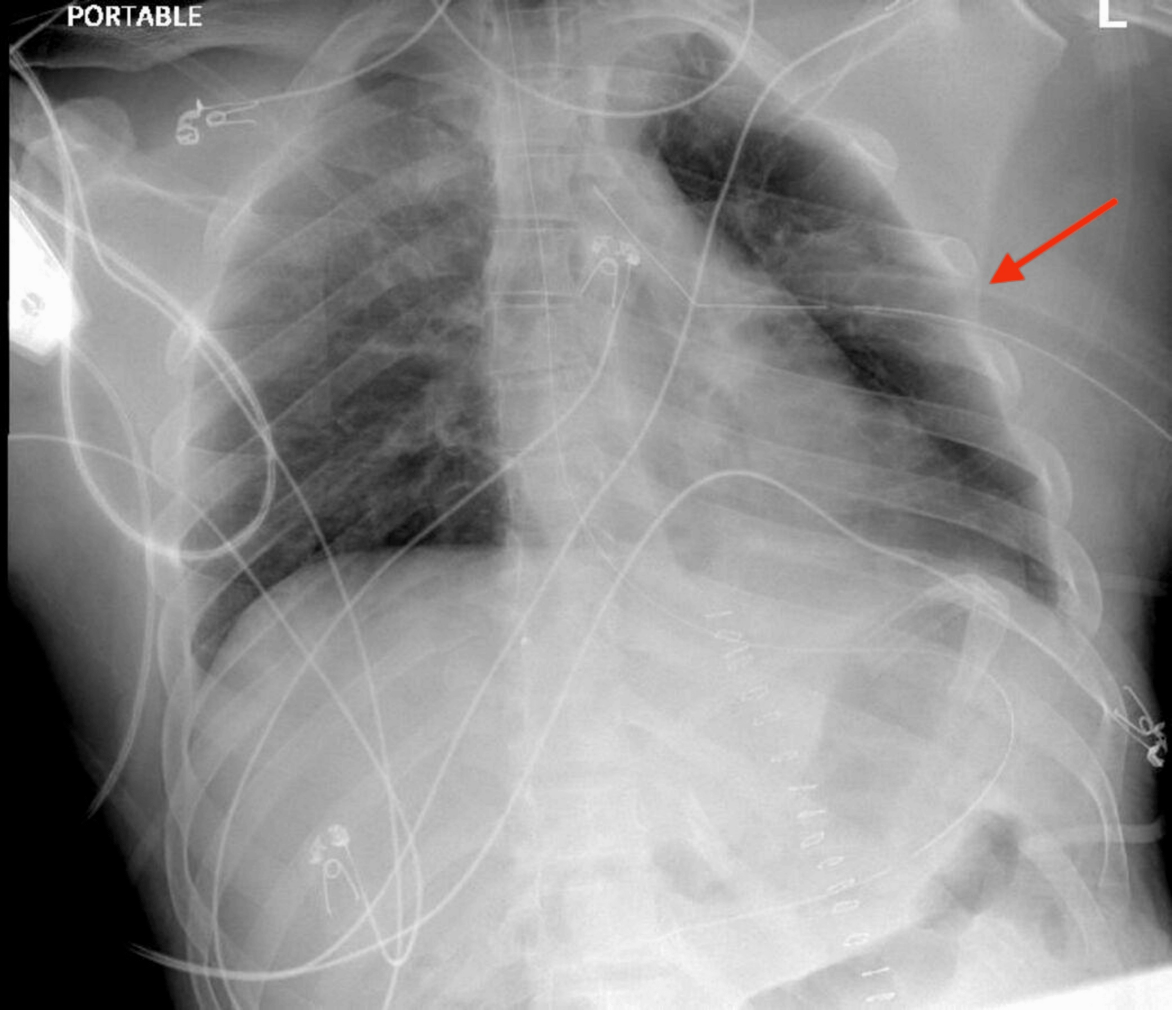
Chest X-ray showing the left chest tube properly placed to evacuate left hemothorax (red arrow).

A chest tube was subsequently placed as visible in Figure 2. He was extubated on post-operative day 1 and underwent left thoracotomy, lung decortication, repair of diaphragmatic injury, and mechanical pleurodesis with subsequent chest tube placement on the third post-operative day. The patient was tolerating the diet, and chest tubes were removed.

**Figure 3:**
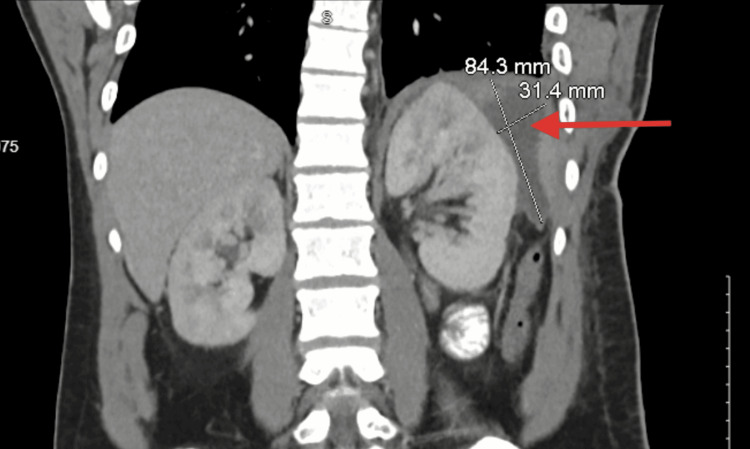
CT abdomen/pelvis showing a multiloculated subphrenic and perisplenic collection (red arrow)

**Figure 4:**
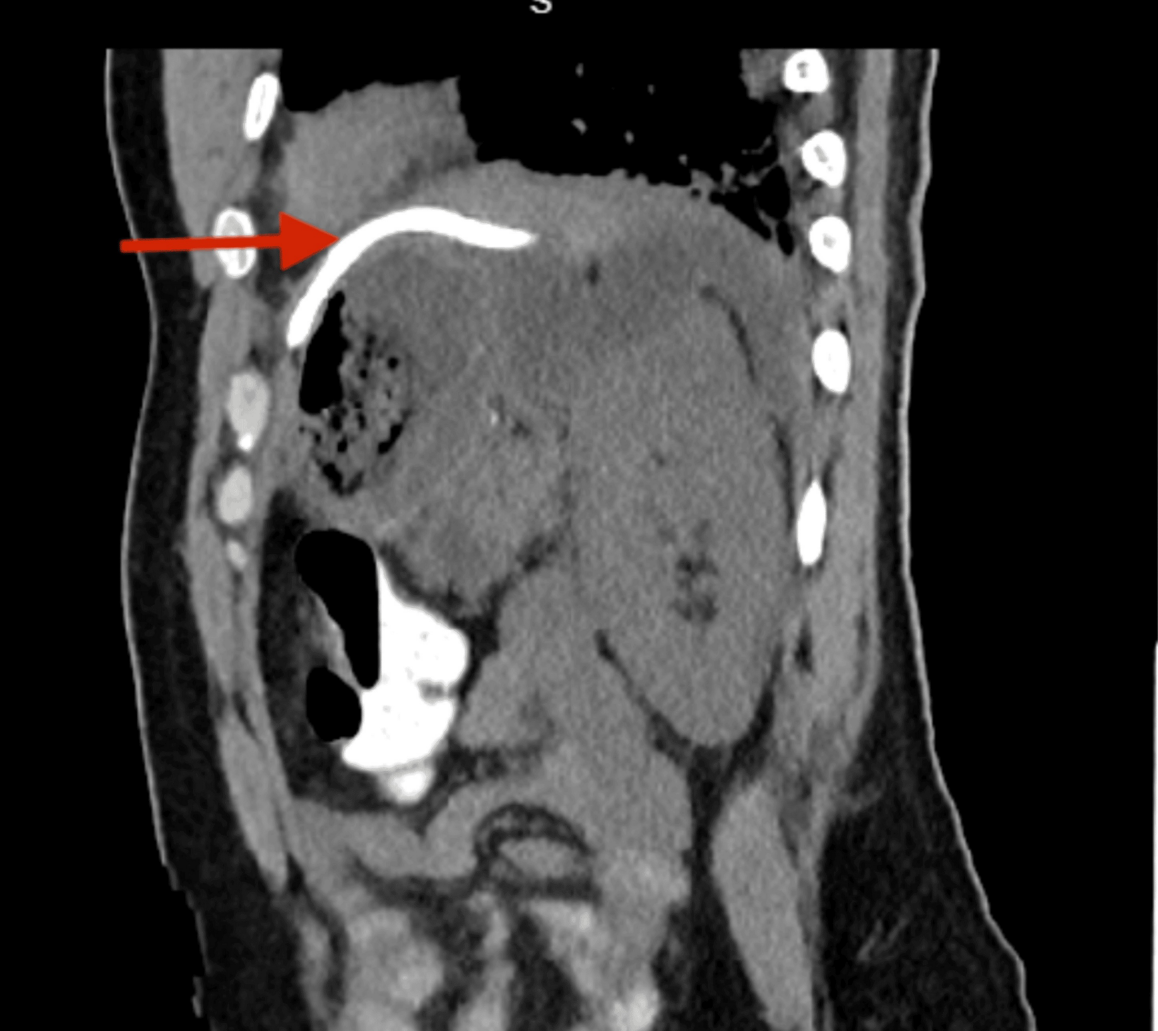
Sagittal view of CT abdomen showing the drain in communication with the collection (red arrow)

On post-operative day 9, the patient developed sepsis. Computed tomography of the abdomen and pelvis demonstrated a multilocular left subphrenic/perisplenic well-circumscribed fluid collection compatible with subphrenic abscess, as demonstrated in Figure 3. The patient was on broad-spectrum antibiotics which included vancomycin, piperacillin/tazobactam, and caspofungin. Interventional radiology was consulted but their team could not drain the collection due to a lack of a window as they would have to go through the pleural cavity. The drain could not be upsized. In a discussion with the patient, we presented the option of a re-exploration vs tPA irrigation through the existing drain that was in communication with the collection as visible in Figure 4. The patient opted for conservative management. We proceeded with 4 mg of tissue plasminogen activator reconstituted and administered through the Jackson Pratt drain with 40 cc of saline, and the drain was clamped for one hour. After unclamping the drain, approximately 150 ml of turbid purulent fluid was immediately returned. Over the next two days, 700 ml of purulent fluid was returned. tPA was instilled once more for a total dose of 6mg with the return of serosanguineous fluid. 

The patient’s clinical status improved, and repeat imaging showed a decreased interval size of fluid collection. The patient was discharged home with a Jackson-Pratt drain in place with oral amoxicillin-clavulanic acid and short-interval follow-up. The final body culture had no growth. At the subsequent clinic visit, the drain output was less than 30 cc/day, and the drain was removed. Repeat imaging revealed near resolution in the collection; the patient returned to work and was given follow-up appointments as needed. 

## Discussion

Intra-abdominal abscesses commonly occur in the setting of bowel perforation, anastomosis leaks, or trauma. They develop as a result of the confluence of multiple variables, such as the existence and location of a lesion (in cases of trauma), predisposition to infection, treatment modality, and contamination of the abdominal cavity [5].

Given that infection is the most common cause of death in patients who survive for longer than 48 hours in trauma settings, management of the post-operative complication is of increased importance to improving outcomes in trauma surgeries [5]. However, prevention of this entity should be a priority. A study performed over four years using ertapenem as a standardized protocol for antimicrobial prophylaxis in patients undergoing trauma laparotomy showed a statistically significant decrease in the incidence of surgical site infections in the post-intervention cohort; though it was not the same for other types of deep/organ infections, it is worth considering in trauma patients [6]. 

The development of an intra-abdominal infection is associated with multiple risk factors. A prospective cohort study showed the abdominal trauma index >24, abdominal contamination, and ICU admission as independent risk factors for the development of this entity after a multivariate logistic regression analysis [7]. 

Regarding management, the most recent literature considers percutaneous CT-guided drainage the gold standard for the management of intra-abdominal abscesses [5]. When first introduced, percutaneous drainage of intra-abdominal abscesses had been shown to have a success rate of up to 86% [5,8]. In most recent studies, over 90% cure rate can be accomplished with percutaneous drainage [9]. While percutaneous drainage offers high success rates with low morbidity, it depends on both the availability and comfort level of the interventional radiologist in accessing the abscess. Moreover, interloop abscesses and proximity to large blood vessels or organs such as the spleen or pancreas can make percutaneous drainage dangerous [10]. In the patients for whom percutaneous drainage of an intra-abdominal abscess does not yield improvement or is not feasible due to lack of an appropriate window for drainage, open or laparoscopic surgical drainage is indicated. Multiple complications can arise from a re-exploration, especially if the time interval from the initial surgery is short. The complications and the desire to avoid unnecessary surgical procedures, if possible, make the viability of other modalities of treatment an enticing prospect. 

Such was the case in this presentation that the patient was unable to undergo percutaneous drainage due to the absence of a viable window. As the patient already had a Jackson-Pratt drain in place due to a pancreatic injury, administration of a tPA through this drain to aid in the drainage of this collection was proposed. After serial administrations of alteplase, the patient began to improve clinically, and serial CT scans of the abdomen revealed a decreasing collection size. The Jackson-Pratt drain was subsequently removed.

Administration of tPA through an abdominal drain has been used in rare instances to address abdominal abscesses and has been shown to be effective in achieving abscess resolution. For example, in a randomized trial in which patients with loculated abdominopelvic abscesses received either tPA or saline solution, abscess resolution was achieved in 80% of the patients in the tPA arm versus 33% in the normal saline arm [11]. 

Another retrospective study reviewing 46 abscesses treated with tPA after incomplete resolution with initial catheter placement showed that 41 abscesses achieved complete resolution, with the remaining five requiring surgical intervention [12]. The successful use of tPA to aid in the drainage and resolution of this intra-abdominal abscess lends support to the viability of this method as an alternative to surgical drainage in patients with a contraindication for percutaneous drainage. The dosing was extrapolated from intra-pleural use [13]. Optimal dosing protocols remain to be defined in time. 

## Conclusions

Intra-abdominal abscesses represent a relatively frequent complication in trauma patients who have undergone laparotomy. Although the gold standard management for this condition is CT-guided drainage, situations may arise where this procedure is contraindicated, such as when the location of the collection hinders access. The use of tPA irrigation as a viable alternative for abscess drainage prior to surgical drainage has the potential to significantly reduce the need for unnecessary surgical procedures, reducing hospital stay length as well as the associated morbidity and mortality. 

## References

[1] Collins G, Allaway MG, Eslick GD, Cox MR (2020). Non-operative management of small post-appendicectomy intra-abdominal abscess is safe and effective. ANZ J Surg.

[2] Gervais DA, Brown SD, Connolly SA, Brec SL, Harisinghani MG, Mueller PR (2004). Percutaneous imaging-guided abdominal and pelvic abscess drainage in children. Radiographics.

[3] Goldberg SR, Anand RJ, Como JJ (2012). Prophylactic antibiotic use in penetrating abdominal trauma: an Eastern Association for the Surgery of Trauma practice management guideline. J Trauma Acute Care Surg.

[4] Zhang S, Ren L, Li Y, Wang J, Yu W, Li N, Li J (2014). Bacteriology and drug susceptibility analysis of pus from patients with severe intra-abdominal infection induced by abdominal trauma. Exp Ther Med.

[5] Ivatury RR, Zubowski R, Psarras P, Nallathambi M, Rohman M, Stahl WM (1988). Intra-abdominal abscess after penetrating abdominal trauma. J Trauma.

[6] Mazuski JE, Symons WJ, Jarman S (2023). Reduction of surgical site infection after trauma laparotomy through use of a specific protocol for antibiotic prophylaxis. Surg Infect (Larchmt).

[7] Morales CH, Villegas MI, Villavicencio R, González G, Pérez LF, Peña AM, Vanegas LE (2004). Intra-abdominal infection in patients with abdominal trauma. Arch Surg.

[8] Gibson DM, Feliciano DV, Mattox KL (1981). Intraabdominal abscess after penetrating abdominal trauma. Am J Surg.

[9] Gee MS, Kim JY, Gervais DA, Hahn PF, Mueller PR (2010). Management of abdominal and pelvic abscesses that persist despite satisfactory percutaneous drainage catheter placement. AJR Am J Roentgenol.

[10] Mehta NY, Lotfollahzadeh S, Copelin II EL (2023). Abdominal abscess. StatPearls [Internet].

[11] Cheng D, Nagata KT, Yoon HC (2008). Randomized prospective comparison of alteplase versus saline solution for the percutaneous treatment of loculated abdominopelvic abscesses. J Vasc Interv Radiol.

[12] Beland MD, Gervais DA, Levis DA, Hahn PF, Arellano RS, Mueller PR (2008). Complex abdominal and pelvic abscesses: efficacy of adjunctive tissue-type plasminogen activator for drainage. Radiology.

[13] Shen KR, Bribriesco A, Crabtree T (2017). The American Association for Thoracic Surgery consensus guidelines for the management of empyema. J Thorac Cardiovasc Surg.

